# College and Hospital Notes

**Published:** 1910-05

**Authors:** 


### COLLEGE AND HOSPITAL NOTES.

---

A municipal tuberculosis hospital, to be known as the J. N. Adam Memorial Hospital, will be established at an early date. The purchase of the site at Perrysburg was completed April 8,

1910, when former Mayor Adam, William A. Douglas, secretary of the hospital trustees, and William B. Frye of the Corporation Counsel's office called on the owners of the farms which go to make up the tract, and Mr. Adam turned over the money—$19,475—excepting $3,400, which represents a mortgage held on one of the farms. The holder of the mortgage prefers it to run its course, which will end in December. This will not interfere with the work of building the hospital.

---

THE Buffalo General Hospital authorities announce that provision has recently been made which permits the use of a number of free beds for cases of obstetrics. Physicians and others are asked to send suitable cases. Each patient will receive painstaking and intelligent attention at the hands of the obstetric staff.

Patients desiring to avail themselves of this provision for free care, should come to the hospital either at the beginning of labor or just before term. Physicians sending cases are invited to be present at the delivery.

For the purpose of preliminary consultation with and examinations of patients a member of the staff will be at the hospital Tuesdays and Fridays at 4.00 P. M.

---

THE District Nursing Association of Buffalo, has been provided with a new home, a fine, large dwelling-house at No. 74 West Utica street, the use of which has been given to the association by Mr. John D. Larkin, rent free. The association has moved into its new quarters and the first meeting of the board was held there recently.

This will afford a large extension of the District Nursing Association's work, for the house is large, there is a good-sized yard in the rear, with plenty of apple trees, and the house and grounds will be used by Miss Jacques for her tuberculosis classes. The place will also be headquarters for the nurses. A housekeeper has been installed and the house has become the main headquarters of the association.

---

THE Children's Hospital Aid Association received substantial suggestions from Dr. Cabot, of Boston, through his lectures on Tuesday, April 12, 1910. The standing committees made excellent reports at the annual meeting showing much progress. The directors chosen for the New Year are:

Mrs, R. B. Adam, Mrs. J. A. Archbald, Mrs. Raymond Bissell, Mrs. Edward H. Butler, Jr., Mrs. F. S. Sidway and Miss Louise Sweeney. The following continue in office: Mrs. G. H. Chisholm, Mrs. N. P. Clement, Mrs. J. C. Dann, Mrs. Evan Hollister, Mrs. Cecil Lightfoot, Mrs. J. S. Otto. A new committee was authorised, called a social service committee, to look after the little patients discharged from the hospital and see that they are given necessary care.

